# Role of Macrofaunal Communities in the Vistula River Plume, the Baltic Sea—Bioturbation and Bioirrigation Potential

**DOI:** 10.3390/biology12020147

**Published:** 2023-01-18

**Authors:** Natalia Anna Miernik, Urszula Janas, Halina Kendzierska

**Affiliations:** Institute of Oceanography, University of Gdańsk, al. Marszałka Piłsudskiego 46, 81-378 Gdynia, Poland

**Keywords:** macrozoobenthos, marine biodiversity, bioturbation, bioirrigation, coastal zone, Baltic Sea, Gulf of Gdańsk, Vistula River plume

## Abstract

**Simple Summary:**

Coastal areas, especially river plumes, are very diverse and dynamic zones where numerous geological, chemical and biological processes take place. This is because fresh water from the river with all substances, including pollutants from the land, mixes with salt water from the sea, creating specific living conditions for the organisms that inhabit the area. These organisms, e.g., macroscopic invertebrates such as mussels or worms, live in the sediment where their movement and feeding activities cause the sediment to mix and allow water to flow through it—these activities are called bioturbation and bioirrigation. Our research aimed to investigate how the structure and functioning of benthic marine ecosystems change with distance from the river mouth. We found that coastal areas are very diverse and host a wide range of organisms that bioturbate and bioirrigate and support sediment transformations relatively deep (up to 15 cm) into the sediment. Farther away from the river mouth, organisms were very scarce and occurred only on the sediment surface and did not burrow into the sediment, so bioturbation and bioirrigation did not take place. The coastal zone is like a hotspot where ecosystem processes and services are intensively reflected, and this is especially important when deeper areas are not functioning properly, as in the Baltic Sea. For this reason, we should consider how we can support the protection and recovery of marine ecosystems.

**Abstract:**

Macrozoobenthos plays a key role in the transformation of inputs from rivers to the sea, such as nutrients, organic matter, or pollutants, and influences biogeochemical processes in the sediments through bioturbation and bioirrigation activity. The purpose of our study was to determine the structure of benthic communities, their bioturbation (BP_C_) and bioirrigation potential (IP_C_), and the vertical distribution of macrofauna in the Gulf of Gdańsk. The study revealed changes in the structure of benthic communities and, consequently, in the bioturbation and bioirrigation potential in the study area. Despite the presence of diverse and rich communities in the coastal zone, BP_C_ and IP_C_ values, although high, were formed by a few species. Both indices were formed mainly by the clam *Macoma balthica* and polychaetes, although the proportion of polychaetes in IP_C_ was higher than in BP_C_. In the deepest zones, the communities became poorer until they eventually disappeared, along with all macrofaunal functions. Both indices changed similarly with distance from the Vistula River mouth, and there was a very strong correlation between them. We also demonstrated that the highest diversity of the macrofauna was observed in the upper first cm of the sediment, but the highest biomass was observed in deeper layers—at a depth of up to 6 cm, and single individuals occurred even below 10 cm.

## 1. Introduction

Coastal zones provide a variety of benefits derived by humans from ecosystem functions and processes. These include nutrient regulation or waste treatment functions, where biota play an important role in storage, recycling or removal of nutrients and compounds [1]. All of these functions help maintain healthy and productive marine ecosystems. Coastal ecosystems with high biodiversity of habitats and benthic communities, especially lagoons, bays and estuaries, play a special role in marine regulatory processes [2,3]. Benthic organisms play a key role in the circulation of chemical elements and nutrients directly by physiological processes such as feeding, respiration and excretion, as well as indirectly by reworking the sediment matrix through bioturbation and bioirrigation [4,5,6,7,8,9]. These activities can be positive for the ecosystem in terms of sediment oxygenation and increasing the surface area available for microbial activity [10,11,12]. Intensive bioturbation or bioirrigation may also lead to the intensification of degradation, transformation or burial of organic matter and contaminants [13,14]. On the other hand, sediment reworking may cause a release of contaminants accumulated in the deeper parts of sediments [14,15]. Thus, bioturbation and bioirrigation play a crucial role in biochemical cycles and production at the seafloor and basin scale [16,17].

At the same time, the coastal zone is particularly exposed to land-based pollution from i.a. increased industrialization, urbanization, agricultural and aquacultural development as well as climate change [18]. Nutrients, organic matter and contaminants from land enter the seas and oceans mainly through surface runoff. Nowadays, river pollution in most populated areas is severe and according to high urbanization future scenarios, about 80% of the global human population is projected to live in sub-basins with multi-pollutant problems [19].

The Gulf of Gdańsk, located in the southern part of the Baltic Sea, is a coastal system with a mixture of fresh and brackish water. Salinity, but also other parameters such as nitrogenous compound and chlorophyll *a* concentrations, change both with distance from the river mouth and with depth [20,21]. Research by Łukawska-Matuszewska et al. [22] showed that sediment toxicity in the Gulf of Gdańsk increases with distance from land, which is associated with an increase in the content of fine sediment fractions, hydrogen sulfide and black carbon, with the latter suggesting anthropogenic contamination of the sediment. The area of the entire Gulf is strongly affected by the Vistula River. It is the longest river flowing into the Baltic Sea, passing through agricultural land, forests and several urban agglomerations [23]. The river has the second largest drainage basin of the rivers flowing into the Baltic Sea (194,000 km^2^, covering 11% of the whole Baltic Sea catchment area). The Vistula River contributes about 90% of the total inflow to the Gulf of Gdańsk [24]. Along with the river’s waters come nutrients, organic matter and various pollutants: heavy metals, organic pollutants, including pharmaceuticals and emerging contaminants [25,26,27]. In addition to the Vistula River, there are other sources of pollutants such as dozens of watercourses, ports, industry, wastewater treatment plants, atmospheric deposition or disturbed sediment [26]. All these compounds reaching the sea can affect the structure and functioning of the ecosystem, while at the same time the presence of organisms such as zoobenthos can help process these compounds. To understand the role of the benthic fauna in these processes, it is necessary to determine how benthic animals are distributed in the vicinity of the Vistula River and how they function.

There is a strong need for indices that demonstrate the decline in ecosystem functioning under anthropopressure and improvement during sustainable ecosystem-based marine management [28,29]. This is due, i.a. to the demand for measures to maintain and improve the ecological status of the marine environment in accordance with the Marine Strategy Framework Directive. Existing bioturbation and bioirrigation potential indices can be used as a proxy of ecosystem processes [30,31,32,33]. Basic benthic monitoring parameters (i.e., abundance and biomass), as well as research-based knowledge (or, in many cases, expert knowledge) of benthic fauna traits related to their behavior in the sediment, are used for the calculations. So far, these coefficients have been successfully used and combined with studies of biogeochemical cycles [34], solutes exchange between water and sediment [33,35], studies of anaerobic episodes [36] and apparent redox discontinuity layer (aRPD) [37]. Although these indices appear simple, they carry some limitations related to insufficient knowledge of the activity of individual species and how it changes under the influence of various factors. However, being aware of these limitations, these tools can be applied in both scientific research and environmental monitoring. According to Queirós and colleagues [38], the bioturbation potential index also has limitations, and knowing this can contribute to more informed use of the index as an indicator of benthic function.

A few studies on the role of macrofauna carried out in the Gulf of Gdańsk have addressed the bioturbation potential index (BP_C_) or nutrient fluxes between water and sediments [39,40,41,42]. Studies on the functioning of marine ecosystems in the Gulf of Gdańsk in recent years have also considered the influence of organic matter on the structure and functioning of trophic networks [43] and how organic matter is transformed by organisms [44]. So far, no research has been carried out in the Gulf of Gdańsk on bioirrigation processes. There are also few published studies on the distribution of organisms in the sediment. They mostly contain information on the depth of occurrence of individual macrofauna and meiofauna taxa [45,46,47,48], but only single studies addressed entire benthic communities [40,41,49].

The objective of this study was to determine the structure of benthic fauna as well as the bioturbation and bioirrigation potential of macrofauna in the sediments of the Vistula plume area, in the Gulf of Gdańsk. Furthermore, it was determined quantitatively how this impact of benthic communities varies depending on the proximity of the Vistula River mouth, as well as which species are the most responsible for sediment matrix reworking in the area. In addition, we have made an attempt to investigate the vertical distribution of macrofauna taxa, detailing their maximal and typical depth of occurrence in the sediment.

The results presented in this paper will help to demonstrate the zones where, due to the presence of animals in the sediments and their activity, nutrients, organic matter and pollutants carried into the Gulf of Gdańsk by the Vistula River are processed. They will also provide knowledge of the vertical distribution of species in the sediments necessary, among other things, for indices of functionality to assess the functioning of the seafloor and basin. Determining the role of macrofauna will also provide arguments for the protection and proper management of marine areas in estuaries.

## 2. Materials and Methods

### 2.1. Sampling

**Figure 1:**
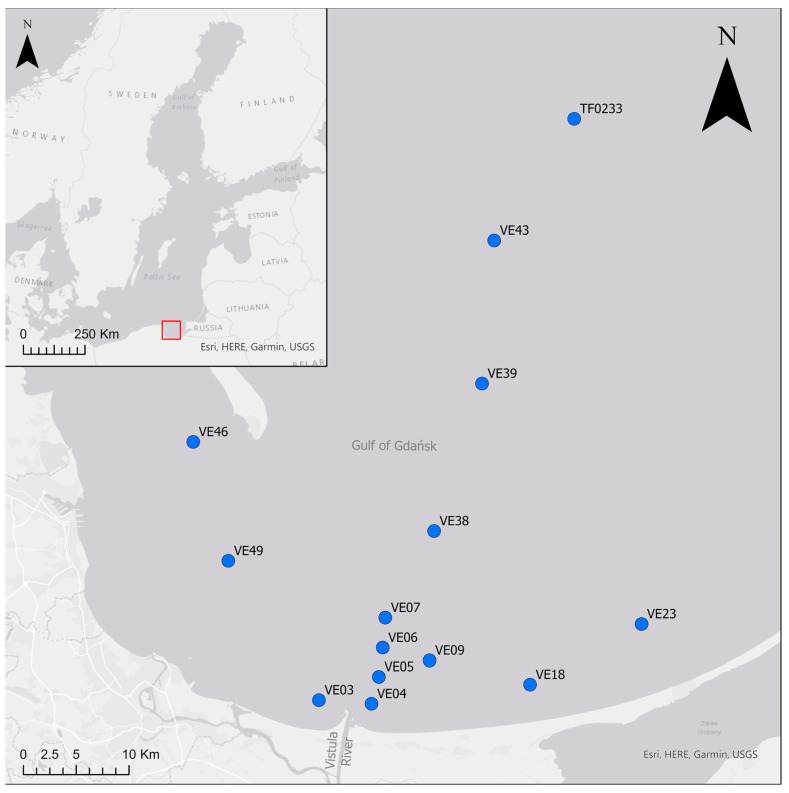
Study area with sampling sites. The red rectangle indicates the location of the study area on a map of the Baltic Sea.

Bottom water, sediment and fauna were collected during two cruises in the Vistula River plume area and along an offshore depth transect in the Gulf of Gdańsk, the Baltic Sea (Figure 1). Samples from 11 sites were collected in July 2014 from the deck of RV Elisabeth Mann-Borgese. In March 2016, three more sites were sampled (VE04, VE06, VE07) during a cruise aboard RV Alkor. Bottom water temperature, salinity and dissolved oxygen (DO) concentration were measured at all sites approximately 0.5 m above the sediment using a Seabird CTD-system with an oxygen SBE43 sensor.

For sediment and macrofauna analysis sediment cores (inner diam. 10 cm) were collected with a multicorer and subsamples of coarse-grained sands were collected from a Haps corer. At each site the upper 10 cm sediment sample for sediment parameters was frozen and prior to all analysis the sediment was dried and homogenized. The organic matter content of the dry sediments was measured as the percentage loss on ignition (LOI) after dry combustion for 8 h at 450 °C and for 5 h at 550 °C for samples collected in March 2016. For grain size analysis, samples were sieved using a shaker and a set of standard test sieves with mesh diameters of 2, 1, 0.5, 0.25, 0.125 and 0.063 mm [50]. Based on a percentage of each class in the total sample mass, sediments were classified by the Udden–Wentworth grain-size scale (after Wentworth [51]).

### 2.2. Macrofauna

For benthic fauna analysis, 3 to 5 replicates were collected at each site, with the exception of station VE49, where only 2 replicates could be collected. Sediment cores were divided into layers: 0–1 cm, 1–3 cm, 3–6 cm, 6–10 cm, 10–15 cm and >15 cm depth. We sifted all layers separately through a 1 mm sieve to separate the macrofauna from the sediment and preserved with 4% formaldehyde until analysis (stored for at least 3 months). In the laboratory, the fauna was sorted and taxa, with the exception of Oligochaeta and *Marenzelleria* spp. polychaetes, were identified to the species level. Taxa were counted and weighed to determine their abundance and biomass (wet mass) per square meter.

### 2.3. Bioturbation Potential (BP_C_) and Irrigation Potential (IP_C_)

To calculate the bioturbation and bioirrigation potential, wet mass (WW) was converted to ash free dry mass (AFDW). The conversion was based on literature data; for bivalves, the coefficients were used for individuals with shells [52,53,54,55]. The Bioturbation Potential Community Index (BP_C_) at individual sites was calculated by summing the bioturbation potentials (BP_i_) calculated for individual taxa [30,36].

(1) $${\mathrm{BP}}_{c}=\sum^{\;}{\mathrm{BP}}_{i}\;\mathrm{where}:{\;\mathrm{BP}}_{i}={\left(\frac{B_{i}}{A_{i}}\right)}^{0.5}*A_{i}*M_{i}*R_{i}$$

where for taxon i: B_i_ is biomass (in ash free dry mass g·m^−2^) and A_i_ is abundance (ind.·m^−2^) at each sample, while M_i_, mobility, and R_i_, sediment reworking, are categorical scores assigned to each species (Table A1).

The Irrigation Potential Community Index (IP_C_) at individual sites was calculated by summing the irrigation potentials (IP_i_) calculated for individual taxa [56].

(2) $${\mathrm{IP}}_{c}=\sum {\mathrm{IP}}_{i}\;\mathrm{where}:\;{\mathrm{IP}}_{i}={\left(\frac{B_{i}}{A_{i}}\right)}^{0.75}*A_{i}*{\mathrm{BT}}_{i}*{\mathrm{FT}}_{i}*{\mathrm{ID}}_{i}$$

where for taxon i: B_i_ is biomass (in ash free dry mass g·m^−2^) and A_i_ is abundance (ind.·m^−2^) at each sample, while feeding type (FT_i_), burrow type (BT_i_) and depth (ID_i_) are scores for the trait categories assigned to each species (Table A1). Exponent 0.5 used in BP_C_ emphasizes the importance of organisms with high density and relatively low biomass, while exponent 0.75 used in IP_C_ emphasizes the activity of organisms with larger sizes but lower densities [33].

### 2.4. Vertical Distrbution of Macrofauna in Sediment

The analysis of the vertical distribution of macrozoobenthos in the sediment to determine the maximum burial depth of each taxon and the entire community was performed for both the abundance and biomass of organisms from 51 cores. To present the vertical distribution, the benthic macrofauna abundance and biomass measured in separate sediment layers were recalculated per 1 dm^3^ volume. The burial depth data were averaged for all cores in which a given taxon occurred. The percentage of individual taxa abundance and biomass (90%) in the studied layers was indicated to determine the typical depth of occurrence.

### 2.5. Data Analysis

Principal Component Analysis (PCA) was carried out to determine the relationship between physicochemical conditions in bottom water and surface sediments, and the variability between the sites. A matrix with normalized data on bottom water temperature, salinity, dissolved oxygen concentration and organic matter content in surface sediments was used in statistical analysis. Environmental parameters were strongly correlated with the depth of the basin i.e., salinity (Pearson’s r = 0.95), DO (r = −0.79), type of sediment (r = 0.86) and LOI (0.65).

Prior to biological data analysis, the biomass at each sampling site was averaged and square root transformed. Cluster (Bray–Curtis similarity) and SIMPROF analysis was used to determine the similarity of macrofauna samples. The SIMPER procedure was applied to identify species responsible for similarities/differences in macrozoobenthic communities between the analyzed sites [57]. Biota and Environment matching analysis (BEST BIO-ENV) was performed to determine the effects of temperature, salinity, DO concentration in bottom water and organic matter content in surface sediment on the formation of benthic fauna communities. Distance-based linear models (distLM) were used to examine the effects of environmental variables on biomass, maximum burrowing depth, BP_C_ and IP_C_ [58]. First, the relationships between the variables were examined and oxygen concentration was excluded from the analysis as being strongly correlated with salinity (Pearson’s r = −0.84). The following three environmental variables were selected: temperature, salinity and LOI and log(x + 1) transformation was used before analysis. Stepwise selection and the AICc stopping criterion were used in distLM to investigate the role of environmental variables in predicting biological traits of macrozoobenthos. Resemblance matrices were based on the Euclidean distance similarities between the sites. The results of marginal tests indicate the proportion of the variation the predictor accounts for on its own, while the results from the sequential test indicate the proportion added by the predictor to the cumulative total proportion explained. The statistical analyses were computed in PRIMER v6 & PERMANOVA +. Maps with results were prepared using Arc GIS Pro 2.9.0, ESRI Inc., Redlands, California, the United States of America.

Data from individual cores were used to analyze the relationship between the biological parameters. The relationship between bioturbation and bioirrigation potential indices (calculated using WW and AFDW, and two different exponents in the case of IP_C_) and the number of taxa, abundance, biomass and maximum burrowing depth were determined by Spearman’s rank correlation test. In addition, IP_C_ values obtained when considering the maximum burrowing depth of macrofaunal individuals in the sediment observed in this study were also compared with those assumed based on the literature and expert knowledge. Prior to the statistical analysis, the normality of the data was tested (Shapiro–Wilk test *p* < 0.05).

## 3. Results

### 3.1. Environmental Conditions

Bottom water temperature at the surveyed sites was relatively uniform (below 6.2 °C), except for sites VE03, VE05, and VE18, which were surveyed in the summer season, above thermocline (Table 1). Bottom water salinity was generally higher in the deeper parts and reached 12.7 in the Gdańsk Deep (site TF0233). The opposite situation was observed for dissolved oxygen concentrations in bottom water. Oxygen conditions were above 4.68 mL·dm^−3^ at the shallow sites, but oxygen deficiency was observed in the deepest part—below 3.41 mL·dm^−3^, and the two deepest sites (VE43 and TF0233) showed hypoxia (DO < 2 mL·dm^−3^). Sediment variability was fairly typical for the coastal areas. The shallow sites were characterized by the presence of medium and fine-grained sands, while deeper sites were dominated by clay and silt.

**Figure 2:**
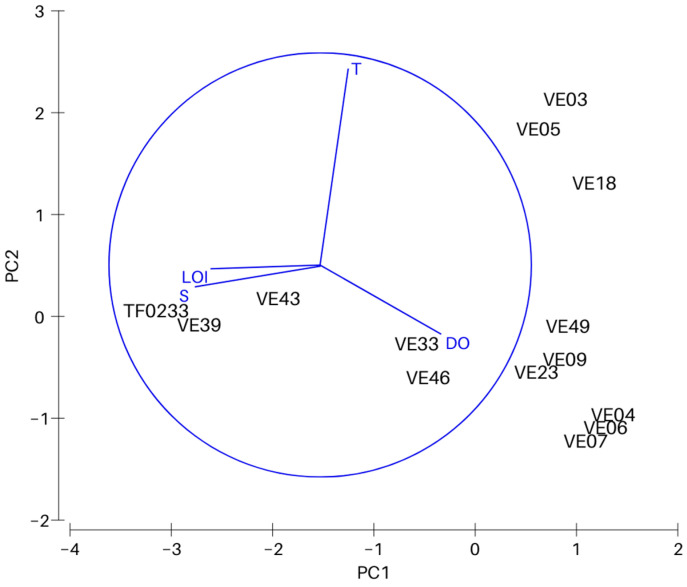
Results of principle component analysis (PCA). Variables included in the PCA are bottom water temperature (T), salinity (S) and oxygen concentration (DO), and organic matter content in the surface sediments (LOI).

PCA analysis was conducted to determine the effect of four physicochemical parameters on the variability between the sites (Figure 2). The first principal component explains 59.6% (eigenvalue 2.39), and together with the second principal component (eigenvalue 1.08) a total of 86.6% of the total variation (Table 2). Salinity with a coefficient of −0.612 has the largest contribution to the distribution along the PC1 axis. The distribution along the PC2 axis was most significantly affected by bottom water temperature (coefficient 0.939).

### 3.2. Macrofauna

The study revealed the presence of a total of 23 macrofaunal taxa in the Gulf of Gdańsk. Taxa with the highest frequency in the Vistula estuary (above 70%) were the bivalve *Macoma balthica*, Oligochaeta, the polychaetes *Bylgides sarsi*, *Marenzelleria* spp., *Pygospio elegans*, as well as the crustacean *Corophium volutator* and the gastropod *Peringia ulvae* (data not shown). The biodiversity of benthic organisms decreased with depth—from 16 taxa at site VE05 to no organisms in the Gdańsk Deep. The main factors determining the structure of macrozoobenthos biomass were salinity and oxygen concentration in the bottom water (BIOENV, r = 0.74).

**Figure 3:**
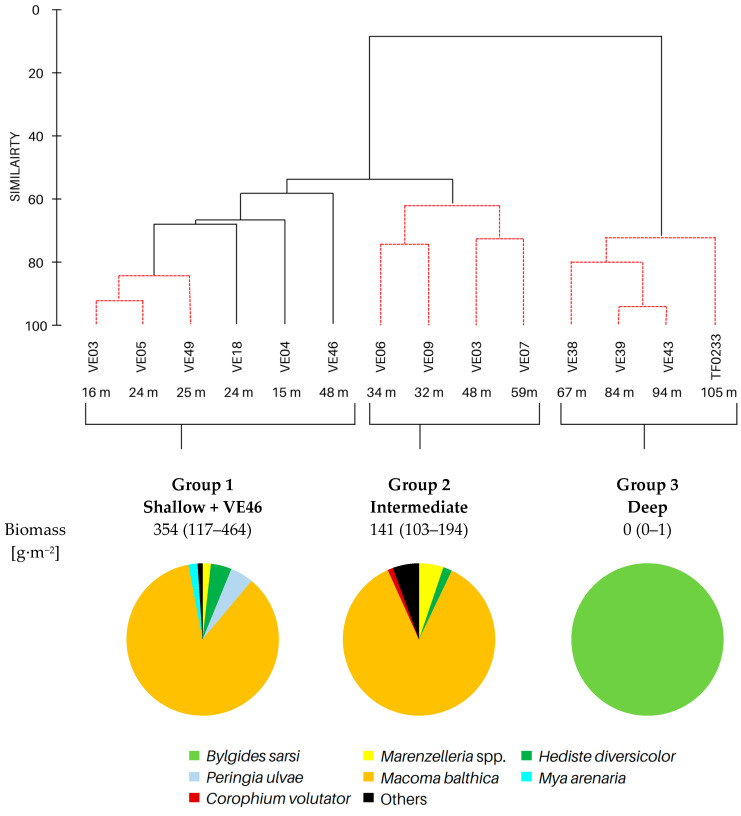
Groups of sites distinguished by cluster analysis on the basis of taxonomic composition and biomass (data transformation: √): **top**—cluster similarity of the study sites; **bottom**—contribution of taxa in macrofaunal biomass in three groups of sites.

Based on cluster and SIMPROF analysis, three groups of sites were distinguished with respect to the biomass of the identified macrofauna taxa (Figure 3). In both group 1 and group 2, *M. balthica* was the most dominant species in the biomass and significantly contributed to the similarity of biomass in both groups (contribution to the total biomass of 67% and 79%, respectively). In addition, species that contributed to the similarity in group 1 were *Hediste diversicolor* (11%), *P. ulvae* (9%) and *Mya arenaria* (8%). Other taxa that accounted for the similarity between sites in group 2, in addition to *M. balthica*, were *Marenzelleria* spp. (7%) and *Halicryptus spinulosus* (5%). In addition, group 3 comprised the deepest sites, where only polychaetes represented by the species *B. sarsi* were observed. The average dissimilarity between group 1 or group 2 and group 3 was >99%. In both cases, *M. balthica* accounted for the highest proportion of dissimilarity (>54%).

All the biological parameters studied reached the highest values at the shallow sites and site VE46, and their values gradually decreased in subsequent groups with increasing depth (Table 3). Benthic communities at the shallow and intermediate sites were characterized by high taxonomic diversity of macrofauna. The highest values of density and biomass of macrofauna were observed at the shallow sites and decreased with depth. Similarly, the values of the BP_C_ and IP_C_ indices decreased, with the values of both indices being lower by half at the intermediate sites compared to the shallow sites.

**Figure 4:**
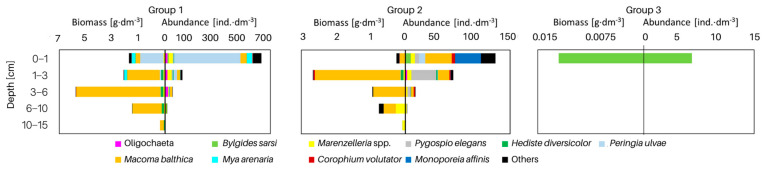
Vertical distribution of macrofaunal taxa deep into the sediment in each group of the sites shown in Figure 3. The scale of abundance and biomass differs for individual groups.

The vertical distribution of organisms in each group differed in terms of both abundance and biomass (Figure 4). In all groups of sites, the largest number (>62%) of organisms was found in the shallowest layer of sediment. This was also the only layer in group 3 containing organisms. However, the maximum biomass of organisms was observed in the deeper sediment layers—as much as 42% of the biomass at the sites from group 1 was found in the 3–6 cm sediment layer, and in group 2, organisms were found in the shallower layers—almost 60% of the biomass was found in the 1–3 cm sediment layer. This is due to the dominance of *M. balthica* in the infaunal biomass.

**Figure 5:**
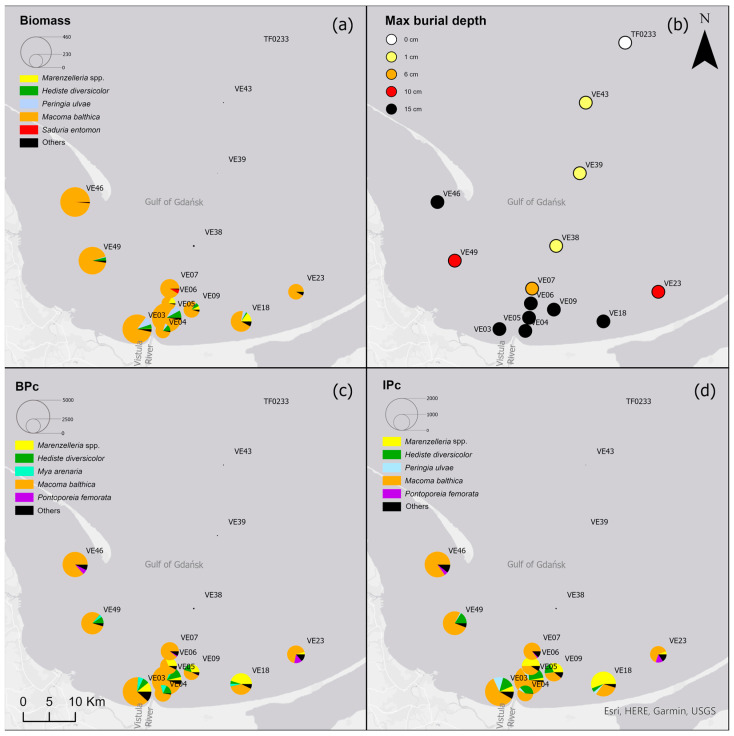
(**a**) Biomass [g∙m^−2^]; (**b**) maximum burial depth of organisms [cm]; (**c**) bioturbation potential index (BP_C_) and (**d**) bioirrigation potential index (IP_C_), and the proportion of taxa in the values of each parameter in the Gulf of Gdańsk.

*M. balthica* accounted for the largest proportion of biomass at all but the deepest sites (Figure 5) (Table A2). The biomass was also composed of *Marenzelleria* spp., *P. ulvae* and *H. diversicolor*. Only epifaunal *B. sarsi* was observed at the deepest sites. The coastal sites were characterized by the occurrence of taxa such as *Marenzelleria* spp. and *H. diversicolor*, which burrow to a depth of 15 cm. With the distance from the Vistula River, fewer taxa were observed burrowing deeper into the sediment.

Both the bioturbation potential index and the bioirrigation potential index followed the distribution of biomass, with the highest values in the shallow areas and in vicinity of the Vistula River mouth, and lower values in the deep area and no bioturbation activity in the Gdańsk Deep. BP_C_ and IP_C_ at all (except the deepest) sites were mainly formed by *M. balthica*. At the shallow sites, the polychaetes, *M. arenaria* and *Pontoporeia femorata* contributed relatively significantly to the formation of BP_C_, while at VE18 it was mainly formed by *Marenzelleria* spp. In the formation of IP_C_, *Marenzelleria* spp. contributed more than other taxa at several sites (VE18, VE06, VE09). The highest BP_C_ (5001) and IP_C_ (1958) values were recorded at site VE05. 

Among environmental parameters, salinity (and highly correlated DO) was the most important predictor, explaining more than 44% of the variability in biomass, burial depth, BP_C_ and IP_C_ (Table 4). Salinity (and highly correlated DO) and temperature, and in the case of BP_C_ also LOI, explained more than 80% of the data variation in BP_C_ and IP_C_.

There are strong positive correlations between bioturbation and bioirrigation potential indices, as well as between them and key characteristics of benthic communities, i.e., the number of taxa, abundance, biomass as well as maximum burrowing depth (Table 5). Similarly strong and significant relationships exist between BP_C_ and IP_C_ calculated from differently presented biomass data (WW and AFDW), as well as when comparing IP_C_ calculated from benthic fauna burial depth data obtained in this study with the index using the literature data. 

**Figure 6:**
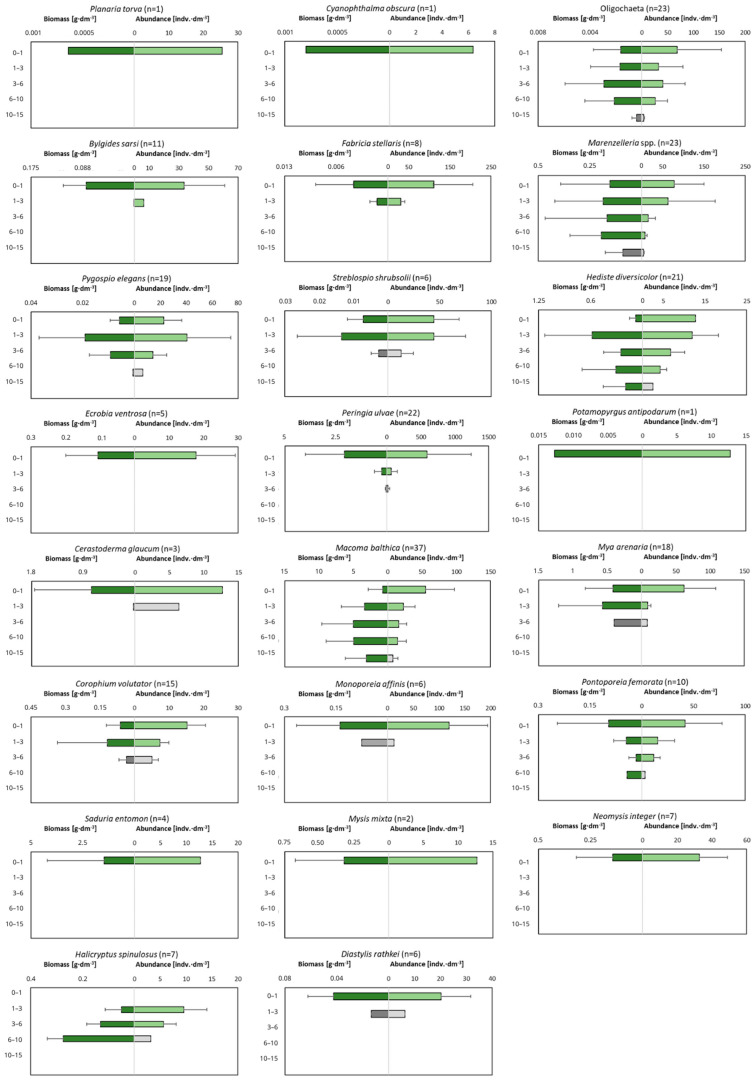
Depth of occurrence of individual macrofaunal taxa in the sediment. Green color indicates to what depth 90% of all organisms are observed. n is number of cores in which particular taxa was observed.

The examination of the macrofauna in different layers of the cores showed that the sediments are inhabited to a depth of 15 cm (Figure 6). All the studied taxa, with the exception of *H. spinulosus*, are observed in the shallowest layer of sediment. For some taxa (*Planaria torva*, *Ecrobia ventrosa*, *Potamopyrgus antipodarum*, *Saduria entomon*, *Mysis mixta* and *Neomysis integer*), this is the only layer of occurrence. Few—especially polychaetes—are observed in the deeper sediment layers, and their dominant abundance and biomass occurs in the 3–6 cm and 6–10 cm layers. The deepest recorded taxa are the polychaetes *Marenzelleria* spp. and *H. diversicolor*, the clams *M. balthica* and Oligochaetes, which were found in the layer up to a maximum of 15 cm deep into the sediment.

## 4. Discussion

### 4.1. Conditions of Bottom Water and Sediments and Their Impact on Macrozoobenthos

Coastal areas, such as estuaries, lagoons and bays, are dynamic environments with gradients of freshwater and seawater flows, representing transition zones between land and sea [3,59,60]. Gradients in physicochemical parameters of bottom water and surface sediments are typical for the Gulf of Gdańsk [41,42,61] [this research]. As the depth of the basin increases, salinity increases, DO decreases, while the proportion of the finest fraction, organic matter content and hydrogen sulfide concentration increases. The area of the Vistula outflow is characterized by the presence of increased amounts of organic matter and nutrients supplied with river runoff [62,63,64,65]. Organic matter and nutrients, as well as contaminants on their way from land to open sea are transformed, retained or removed by biota or moved unchanged to the offshore areas of the Baltic Sea [26,44,66,67,68]. 

The conditions prevailing in the bottom water and sediments affect the distribution and species composition of macrozoobenthos. In the case of benthic communities inhabiting the seabed of the Gulf of Gdańsk, the factors that had the greatest impact on biomass structure, macrofauna burial depth and indices of bioturbation and bioirrigation potential were conditions such as salinity and oxygen concentration in the water above the bottom, and factors strongly related to these, such as sediment conditions. It is known that as oxygen conditions in the water above the seabed deteriorate, the concentration of toxic hydrogen sulfide in the sediments increases [41,69,70,71]. 

### 4.2. Macrozoobenthos

The present study revealed the presence of 23 taxa of the benthic macrofauna in the study area. The results were similar to those obtained during other macrozoobenthos studies conducted in the Vistula estuary [39,41,72,73]. The greatest diversity of benthic organisms was observed in the coastal zone, where the density was dominated by *P. ulvae*, a gastropod species typical of the coastal zone in the Baltic Sea, while in the deeper zones the species composition of the benthic community shifted and the abundance was dominated by *P. elegans* and *M. balthica*, species also common in the Baltic Sea. The biomass in all but the deepest zones was dominated by *M. balthica*.

The highest number of taxa was observed at some distance from the Vistula estuary (at a depth of 16–24 m). Relatively few taxa were found at the shallowest site (15 m depth), due to the fact that the estuary is highly dynamic and the material carried by the river forms an unstable and easily eroded substrate, unfavourable to macrozoobenthos development [72,73,74,75]. Although organic matter carried with river runoff constitutes food resources for macrofauna [44], it can also cause benthic organisms to become covered and buried, leading in extreme cases to the complete disappearance of benthic macrofauna in a given area [76]. As the depth of the water body increases, both the taxonomic diversity and the biomass of the macrofauna decreases. At the deepest sites, the macrofauna is either absent or represented by single individuals of the surface-living, semi-pelagic polychaete *B. sarsi*. The reason for this is the decomposition of large amounts of organic matter accumulating on the bottom and stable stratification in the deeper area, which leads to oxygen deficiency or anoxia at the bottom and occurrence of hydrogen sulfide in the surface sediments [69]. These conditions adversely affect the behaviour, physiological processes, fitness of the benthic fauna, and consequently lead to a loss of functions performed by the benthic fauna [36,77,78]. Such a loss of biodiversity can result in reduced resistance of the environment to stress [79]. 

### 4.3. Bioturbation and Bioirrigation

The research carried out has shown that while the zoobenthos biomass in the Vistula estuary is completely dominated by *M. balthica*, the use of bioturbation and bioirrigation potential indices reveals the role of other species, i.e., those whose biomass is not large but it is known from experimental studies that their activity can significantly affect biogeochemical processes [80,81]. The benthic communities described in this study are characterized by their high bioturbation and bioirrigation potential in the coastal region. This is where their impact on various compounds is most likely to be greatest. *M. balthica*, whose intensive bioturbation and bioirrigation activity is relatively well studied, had the largest contribution to the indices [80,81]. Polychaetes of the genus *Marenzelleria* also contributed relatively significantly to the bioirrigation potential index. Experimental studies have shown that this species is an extremely effective bioirrigator and bioturbator [6,48,82,83]. In situ experiments in the Vistula plume showed a significant increase in nutrient fluxes from sediments inhabited by macrofauna, with the greatest impact observed in the presence of polychaetes [39]. In previous studies, a comparison between bioturbation and bioirrigation potential indices maps showed a very similar pattern, but also some differences [56,84]. For example, differences on a spatial scale were found in the German Bight, with higher IP_C_ scores in areas where sessile or semi-sessile species (i.e., *Lanice conchilega* and *Notomastus latericeus*) were particularly abundant [56]. In the Vistula estuary, such a difference is apparent only for one site (VE18), where higher IP_C_ values compared to BP_C_ are due to the abundance of *Marenzelleria* spp.

The present study demonstrated a strong positive relationship between the two indices and their strong correlation with both the maximum burrowing depth of macrozoobenthos, the number of taxa, as well as the abundance and total biomass of macrozoobenthos. Interestingly, there was virtually no difference in these relationships regardless of how the calculations were made (i.e., wet or ash free dry mass). In an earlier study conducted in another region of the Baltic Sea, the authors found no relationship between the bioirrigation index and the number of taxa [84]. According to Queirós et al. [38], BP_C_ was found to be a good predictor of bioturbation distance (average distance travelled by a sediment particle). However, it was found unsuitable for determining other attributes of infauna, such as bioturbation activity, bioturbation depth or diffusion transport. In addition, the index also appears to be a better predictor of community-level estimates, rather than those for individual species. Statistical models using experimental results showed that BP_C_ explained a considerable amount of variance in oxic processes, i.a. oxic mineralization, total N mineralization, and nitrification [85]. Few studies have also been conducted to determine the correlation between bioirrigation potential index values and actual bioirrigation. However, the results of these studies are inconclusive and require further research. A study by De Borger et al. [86] showed that IP_C_ correlates more strongly with burrow ventilation depth than with ventilation rate. The correlation between IP_C_ and irrigation rate was not confirmed by Toussaint et al. [85]. 

The present study did not use the bioirrigation index (BIP_C_) proposed by Renz et al. [32], the scoring system of which additionally takes into account the distinction between the advection and diffusion system performance. The use of this index would result in higher values for free living species and species living in burrows as well as facultative deposit/suspension feeders in advective sediments. Furthermore, it would result in even higher values of bioirrigation potential in the coastal zone, where the advection system dominates, and an even higher proportion of *M. balthica* or polychaetes *H. diversicolor* and *Marenzelleria* spp. in the index for this zone. At the deeper sites where diffusive sediments occur, bioirrigation potential would be much lower than in the coastal zones. The system by Renz and co-workers [32] would emphasize the variability of the bioirrigation index in the Gulf of Gdańsk and the gradual loss of this function in the environment with increasing depth of the water body. Both approaches to the determination of the bioirrigation potential index are certainly worth testing in further studies, especially those combining studies of benthic assemblages, including functional indices, with experimental studies of the impact of macrofauna on biogeochemical processes, or measurements of animal activity. 

The indices used provide only a simplified approximation of the potential capabilities of benthic communities. Bioturbation and bioirrigation are dynamic and complex activities performed by those organisms. They are determined by a number of factors that affect the biological functions of these animals. BP_C_ was observed to follow the seasonal pattern in seawater temperature, with the highest values in summer and autumn [38]. However, it should be kept in mind that temperature and food availability have the potential to impact bioturbation and bioirrigation intensity, as these factors affect physiological processes of benthic species. Studies conducted on the polychaete *Alitta virens* showed that sediment reworking processes could be affected by both low and high temperature, with the lowest bioturbation intensity under low temperature [87]. Oxygen depletion may also change the activity of animals in the sediment, thus affecting bioturbation and bioirrigation. Depending on the oxygen concentration and exposure time, these conditions can result in, for example, an increase in burrow ventilation, a decrease in animal activity or no activity at all [88,89,90].

### 4.4. Burrowing Depth

The burrowing depth of organisms provides, among other things, an indication of the depth to which they can affect the conditions and processes in the sediments. In the present study, most of the organisms (>62% of all individuals) inhabited the shallowest layer of sediment (0–1 cm). The maximum biomass of organisms can be found in the deeper layers of sediment—deeper layers (3–6 cm) at the shallowest sites and slightly shallower layers at the intermediate sites (1–3 cm). A similar distribution of organisms deep into the sediment was observed in earlier studies conducted in the Gulf of Gdańsk—the highest abundance of organisms was found in the shallowest layer of the sediment and it decreased with depth [40,41]. In contrast to the abundance, the biomass of organisms in the shallow water zone did not decrease with depth and its distribution was more varied—the highest biomass was usually observed in deeper layers, i.e., up to 6 cm into the sediment [40].

However, even organisms living on the sediment surface can play an extremely important role by being active in disrupting the diffusive boundary layer, which improves the oxygen conditions of the sediment [91]. Few organisms, i.e., bivalves and polychaetes, burrow naturally into the sediment and are rarely present on its surface [83,92], and their typical burrowing depth is 3–10 cm. The maximum depth of occurrence of a given taxon depends on the ability of the organism to contact the sediment surface, for example, the burial depth of *M. balthica* depends on the length of the clam’s siphon, which is often also related to the size and age of the organism [46]. Our research showed the occurrence of *M. balthica* below a depth of 10 cm, which is also the maximum depth at which the bivalves bioturbate and bioirrigate the sediments. Other deep burrowing species—from the genus *Marenzelleria*—were found up to a depth of 15 cm, but some scientists indicate that these species can burrow as deep as 35 cm [93]. These deep burrowing organisms, such as polychaetes, form burrows that enable water transport in the sediment and aerobic chemical reactions in the deeper layers, as well as affect nutrient cycling [83,94,95].

The vertical distribution of organisms is determined by environmental factors. Organisms change the depth of their occurrence seasonally [46], e.g., *M. balthica* has been shown to burrow deepest in winter and remain shallowly buried in the sediment during the summer season. Oxygen deficiencies and hydrogen sulfide cause the animals to move to the sediment surface or they become periodically inactive [92,96,97]. While animals are present in the sediment, their functions may be temporarily impaired.

## 5. Conclusions 

Our research has shown changes in the structure and functioning of benthic communities with increasing distance from the Vistula River mouth. Coastal zones are characterized by relatively high biodiversity and great burrowing depth of macrofauna, as well as high bioturbation and bioirrigation potential of benthic communities. However, this activity disappears in deep zones with the absence of benthic organisms. The lack of bioturbation and bioirrigation means there is no support for biogeochemical transformation by the macrofauna in the deep zones. In the study area, only a few species drive bioturbation and bioirrigation—the bivalve *M. balthica* and the polychaetes *H. diversicolor* and *Marenzelleria* spp. Other taxa had a marginal impact. Such a strong dominance of single taxa in performing bioturbation and bioirrigation could lead to instability in ecosystem functioning in the case that these organisms were to disappear as a result of an ecological disaster, environmental degradation or disease. At the same time, these large organisms were the only taxa burrowing deep into the sediment (below 10 cm), and thus the only ones supporting geochemical processes deep in the sediment. To summarize, the coastal zone, unlike the offshore zone, proved to be a hotspot for bioturbation- and bioirrigation-driven processes, which are responsible for the proper functioning of the seafloor and basin. However, very poor functional diversity of the benthic macrofauna in the deepest zones means that we should appreciate and protect coastal zones more efficiently.

## Figures and Tables

**Table 1:** Values of sediment characteristics and environmental variables measured in the bottom waters at research sites.

Station	Temperature [°C]	Salinity	Oxygen [mL·dm^−3^]	Sediment Type	LOI [%]	Depth [m]
VE04	4.2	7.6	8.10	Fine-grained sand	1.25	15
VE03	14.2	7.4	5.65	Fine-grained sand	4.49	16
VE05	12.6	7.4	4.68	Fine-grained sand	4.03	24
VE18	11.2	7.3	5.83	Fine-grained sand	0.91	24
VE49	6.2	7.6	5.93	Medium-grained sand	0.89	25
VE09	5.3	8.0	6.32	Medium-grained sand	0.81	32
VE06	3.8	8.0	8.19	Fine-grained sand	0.88	38
VE23	5.0	8.0	6.47	Sandy silt	4.24	48
VE46	4.6	8.2	5.90	Silt	13.26	48
VE07	3.7	8.0	8.33	Fine-grained sand	3.07	59
VE38	4.5	9.1	3.41	Silt	4.40	67
VE39	5.3	11.2	2.56	Silty clay	18.54	84
VE43	5.8	12.3	1.52	Silty clay	3.18	94
TF0233	5.6	12.7	1.59	Silty clay	15.49	105

**Table 2:** Percentage of variation and coefficients in the linear combinations of variables forming PCs.

Variable	PC1	PC2	PC3
Variation [%]	59.6	26.9	11.7
Temperature (T)	0.128	0.939	0.104
Salinity (S)	−0.612	−0.100	−0.366
Oxygen (DO)	0.576	−0.329	0.366
LOI	−0.527	−0.015	0.849

**Table 3:** Number of taxa, maximum burial depth of macrofauna, mean values: abundance, BP_C_ and IP_C_ (min.–max), contribution of individual taxa to the formation of these parameters, and in each group of sites provided in Figure 3.

	Group 1	Group 2	Group 3
No. of taxa	11 (6–16)	8 (7–10)	0 (0–1)
Max. burial depth [cm]	14 (10–15)	11 (6–15)	0 (0–1)
Abundance [ind.∙m^−2^]	11,030 (3628–30,557)	3188 (2578–3851)	63 (0–127)
	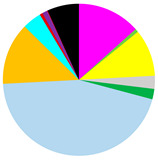	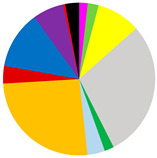	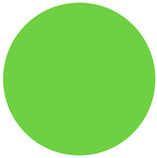
BP_C_	3412 (1576–5000)	1683 (1412–1890)	5 (0–13)
	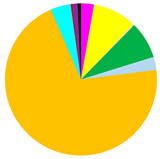	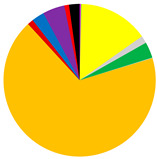	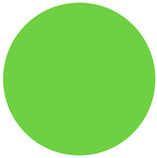
IP_C_	1451 (581–1985)	714 (632–785)	1 (0–4)
	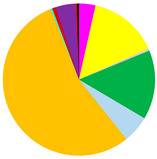	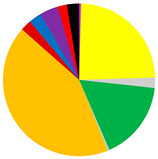	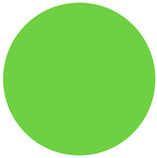
	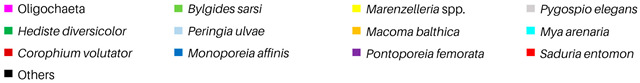		

**Table 4:** Proportion of the variables explaining the distLM model adjustment in marginal and sequential tests for biomass, burial depth, BP_C_ and IP_C_.

	Biomass		Burial Depth		BP_C_		IP_C_	
	Marginal Test	Sequential Test	Marginal Test	Sequential Test	Marginal Test	Sequential Test	Marginal Test	Sequential Test
Salinity	0.442 **	0.442 *	0.644 **	0.644 **	0.529 **	0.529 **	0.564 **	0.564 **
Temperature	0.302 *	0.153	0.03		0.425 *	0.23 **	0.456 **	0.247 *
LOI	0.01	0.1021	0.250		0.027	0.076 *	0.062	
Total		0.697		0.644		0.835		0.811

Significance levels * *p* < 0.05, ** *p* < 0.01.

**Table 5:** Spearman’s correlation coefficients of biological parameters from all sampling sites. Both indices were calculated using different exponents (0.5 or 0.75) and two types of animal biomass—wet formalin mass (WW) or ash free dry mass (AFDW). Significance level for all correlations was *p* < 0.000001.

	$BPc_{WW}^{0.5}$	$BPc_{AFDW}^{0.5}$	$BPc_{AFDW}^{0.75}$	$IPc_{WW}^{0.75}$	$IPc_{AFDW}^{0.75}$	$IPc_{AFDW}^{0.75}$ **IDi lit.**	Max. Burrowing Depth	No. of Taxa	Abundance	Biomass _WW_	Biomass _AFDW_
$BPc_{WW}^{0.5}$		0.999	0.991	0.978	0.982	0.982	0.672	0.756	0.934	0.968	0.970
$BPc_{AFDW}^{0.5}$			0.990	0.990	0.986	0.985	0.678	0.766	0.938	0.965	0.968
$BPc_{AFDW}^{0.75}$				0.984	0.977	0.976	0.663	0.732	0.915	0.989	0.990
$IPc_{WW}^{0.75}$					0.999	0.997	0.708	0.785	0.940	0.980	0.963
$IPc_{AFDW}^{0.75}$						0.997	0.720	0.792	0.942	0.953	0.956
$IPc_{AFDW}^{0.75}$ **IDi lit.**							0.718	0.785	0.935	0.949	0.953
**Max. burrowing depth**								0.730	0.717	0.637	0.637
**No. of taxa**									0.836	0.706	0.706
**Abundance**										0.880	0.883
**Biomass _WW_**											0.999
**Biomass _AFDW_**											

## Data Availability

Not applicable.

## Appendix A

**Table A1:** Categorical scores assigned to each taxon for BP_c_ and IP_c_ indices calculations according to Solan et al. [30], Villnäs et al. [36] Queirós et al. [31] and Wrede et al. [98] (modified), where: Mobility (M_i_): 1, feeding on the sediment surface; limited movement on the sediment surface; sessile; 2, limited movement; 3, slow free movement; 4, free to movement. Reworking type (R_i_): 1, epifauna; 2, surficial modifiers; 3, upward or downward conveyor; 4, biodiffusors. Burrow type (BT_i_): 1, epifauna or internal irrigation (i.e., siphons); 2, open irrigation (i.e., Y- or U-shaped burrow); 3, blind ended burrow. Feeding type (FT_i_): 1, surface filter feeder; 2, predator; 3, deposit feeder; 4, sub-surface filter feeder. Irrigation depth (ID_i_): 1, 0–1 cm; 2, 1–3 cm; 3, 3–6 cm; 4, 6–10 cm; 5, 10–15 cm.

Taxa	BP_c_		IP_c_		
	M_i_	R_i_	BT_i_	FT_i_	ID_i_
*Planaria torva*	1	1	1	2	1
*Cyanophthalma obscura*	3	1	3	2	2
Oligochaeta	3	2	3	3	4
*Bylgides sarsi*	3	1	1	2	2
*Fabricia stellaris*	2	1	3	1	2
*Marenzelleria* spp.	4	4	3	3	5
*Pygospio elegans*	2	2	3	3	3
*Streblospio shrubsolii*	2	2	3	3	2
*Hediste diversicolor*	4	3	2	3	5
*Ecrobia ventrosa*	1	1	1	3	1
*Peringia ulvae*	1	1	1	3	2
*Potamopyrgus antipodarum*	1	1	1	3	1
*Cerastoderma glaucum*	3	2	1	1	1
*Macoma balthica*	3	4	1	3	4
*Mya arenaria*	3	4	1	1	2
*Corophium volutator*	2	2	2	3	3
*Monoporeia affinis*	4	2	3	3	2
*Pontoporeia femorata*	4	4	3	3	4
*Diastylis rathkei*	3	2	3	3	2
*Saduria entomon*	4	2	3	3	1
*Mysis mixta*	4	1	3	3	1
*Neomysis integer*	4	1	3	3	1
*Halicryptus spinulosus*	3	4	3	3	4

**Table A2:** Average (±SD) number of taxa and biomass (g. m^−2^) at the sampling sites of the dominant taxa present (*n* = 2 for sites VE49, *n* = 3 for TF0233, VE09, VE38, VE39, VE43; *n* = 4 for VE04, VE06, VE07, VE18, VE23, VE46 and *n* = 5 for VE03 and VE05).

Site	No. of Taxa	*Marenzelleria* spp.	*Hediste* *diversicolor*	*Peringia ulvae*	*Macoma balthica*	*Saduria entomon*	Others *
VE03	8 ± 4	1.9 ± 2.0	21.7 ± 22.1	42.5 ± 26.1	370.7 ± 227.2	0.0 ± 0.0	14.6 ± 10.1
VE04	5 ± 0	0.0 ± 0.0	16.2 ± 13.0	7.6 ± 8.4	90.1 ± 97.4	0.0 ± 0.0	3.4 ± 2
VE05	8 ± 3	1.1 ± 2.1	35.4 ± 19.3	39.3 ± 8.6	375.5 ± 117.5	0.0 ± 0.0	10.8 ± 11.7
VE06	5 ± 0	18.3 ± 6.8	0.0 ± 0.0	0.1 ± 0.2	81.8 ± 57.9	0.0 ± 0.0	3.2 ± 2.6
VE07	4 ± 1	0.0 ± 0.0	0.0 ± 0.0	0.0 ± 0.0	177.2 ± 77.5	14.0 ± 27.9	3 ± 3.7
VE09	5 ± 4	10.0 ± 8.7	10.6 ± 17	1.1 ± 1.9	109.1 ± 113.5	0.0 ± 0.0	6.1 ± 7.3
VE18	9 ± 2	34.1 ± 30.3	4.6 ± 6.5	12.0 ± 7.4	158.7 ± 59.8	0.0 ± 0.0	18.2 ± 16.4
VE23	6 ± 1	1.1 ± 0.9	0.0 ± 0.0	0.0 ± 0.0	118.0 ± 150.6	0.1 ± 0.1	10.3 ± 7.7
VE38	0 ± 0	0.0 ± 0.0	0.0 ± 0.0	0.0 ± 0.0	0.0 ± 0.0	0.0 ± 0.0	1.8 ± 3.1
VE39	0 ± 0	0.0 ± 0.0	0.0 ± 0.0	0.0 ± 0.0	0.0 ± 0.0	0.0 ± 0.0	0.2 ± 0.2
VE43	0 ± 0	0.0 ± 0.0	0.0 ± 0.0	0.0 ± 0.0	0.0 ± 0.0	0.0 ± 0.0	0.4 ± 0.7
VE46	5 ± 0	0.1 ± 0.1	0.0 ± 0.0	0.0 ± 0.0	458.1 ± 82.1	0.7 ± 1.4	5.8 ± 3.3
VE49	7 ± 0	0.0 ± 0.0	15.9 ± 6.1	3.8 ± 0.8	374.7 ± 183.2	0.0 ± 0.0	10.2 ± 7.1
TF0233	0 ± 0	0.0 ± 0.0	0.0 ± 0.0	0.0 ± 0.0	0.0 ± 0.0	0.0 ± 0.0	0.0 ± 0.0

* Species covered by the category “Others” include the taxa listed in Table A1.
